# Central line change in potential catheter-related bloodstream infection: target for intervention to reduce harm

**DOI:** 10.1186/cc9650

**Published:** 2011-03-11

**Authors:** R Davies, M Lowings, AT Jones, CJ Langrish

**Affiliations:** 1 St Thomas' Hospital, London, UK

## Introduction

Central venous catheterization is routine in critical care, but a potential source of harm. Forty-two per cent of bloodstream infections in England are central-line related [1], at a substantial cost to the health service. Early catheter removal is vital for source control where catheter-related bloodstream infection (CRBSI) is suspected. Furthermore, a model encompassing daily review and removal of unnecessary catheters has been shown to reduce the risk [2]. We studied the time from decision to removal of existing central venous catheters (CVCs), and evaluated potential reasons for delay.

## Methods

This is a retrospective review of practice at a 43-bed medical/surgical ICU at a London teaching hospital, using computerized patient records. All patients requiring a change of CVC over a 2-month period in 2010 were included. Change of CVC was defined as the time from decision to removal of the old CVC, incorporating new CVC insertion. Sepsis was defined as rising inflammatory markers, an impression of local/systemic infection, or emergency (unsterile) insertion. Routine was defined as no signs of infection, usually at 5 to 7 days or if accidentally dislodged/blocked.

## Results

Seventy-eight CVC changes were performed, 45 (57.7%) for sepsis and 33 (42.3%) as routine. The median time to change a septic CVC was 742.5 minutes (106 to 2,038 minutes). The median time for a routine change was 611 minutes (130 to 1,759 minutes). On average, 70% of the time taken to change a CVC involved new catheter insertion. Where the tip position was confirmed with a chest X-ray scan, it took a median of 182 minutes longer (-97 to 946 minutes) to change the CVC. Check X-ray review was documented in 28 (45.1%) of 62 internal jugular/subclavian CVCs and only five X-ray scans resulted in repositioning. Where inotropes/vasopressors were administered, it took a median of 209 minutes longer (106 to 599 minutes) for CVC change. Where coagulation products were administered, it took a median of 168.5 minutes longer (209 to 279 minutes) for CVC change.

## Conclusions

Our data suggest that in our unit the duration of catheter change in the critically ill is a prolonged process, and took longer where potential harm is greatest. Check X-ray scans infrequently result in CVC repositioning, contribute to delays and could be performed after old-CVC removal. We plan to audit the changes we have made, and believe that the timely exchange of old CVCs should be incorporated into models aiming to reduce the impact of CRBSI.
